# Treatment of primary intracranial germ cell tumors: Single center experience with 42 clinically diagnosed cases

**DOI:** 10.18632/oncotarget.10218

**Published:** 2016-06-22

**Authors:** Qun-Ying Yang, Cheng-Cheng Guo, Mei-Ling Deng, Jian Wang, Jing Wang, Fu-hua Lin, Ji Zhang, Xiao-Bing Jiang, Yong-Gao Mou, Zhong-Ping Chen

**Affiliations:** ^1^ State Key Laboratory of Oncology in South China, Department of Neurosurgery/Neuro-Oncology at The Cancer Center of Sun Yat-sen University, Collaborative Innovation Center of Oncology, Guangzhou 510060, China; ^2^ State Key Laboratory of Oncology in South China, Department of Radiotherapy at The Cancer Center of Sun Yat-sen University, Collaborative Innovation Center of Oncology, Guangzhou 510060, China

**Keywords:** primary intracranial germ cell tumor, clinical diagnosis, diagnostic radiotherapy, diagnostic chemotherapy

## Abstract

**Background and Objective:**

Primary intracranial germ cell tumors (GCTs) are a class of heterogeneous tumors. Surgery can quickly relieve tumor compression and provide histological diagnosis. It is very difficult to treat some patients who are unable to be pathologically diagnosed. We aimed to analyze clinically diagnosed GCTs patients.

**Methods:**

Patients clinically diagnosed as primary intracranial GCTs were included in this study.

**Results:**

From 2002 to 2015, 42 patients clinically diagnosed with primary intracranial GCTs received chemotherapy and/or radiotherapy. Patients were assigned to diagnostic chemotherapy group (25 cases), diagnostic radiotherapy group (5 cases) and gamma knife radiosurgery group (12 cases) based on their initial anti-tumor therapy. The 5-year survival rates were 85.8%, 75.0% and 63.6%, respectively. There were no statistically significant difference (*p* value = 0.44). Patients were assigned to the group (30 cases) with secretory tumors and the group (12 cases) with non-secretory tumors based on their levels of tumor makers. The 5- year survival rates were 80.7% and 68.6%, respectively. There were no statistically significant difference (*p* value = 0.49).The major adverse reactions were grade III - IV bone marrow suppression with an incidence of 35.2% and grade II- III nausea/vomiting with an incidence of 45.8%.

**Conclusion:**

Surgical removal of tumor or biopsy is recognized as the most accurate method to determine the pathological property of tumor. But for some patients who can not be pathologically diagnosed, they can receive comprehensive treatments such as chemotherapy combined with radiotherapy, and some of them can still have good responses.

## INTRODUCTION

Primary intracranial germ cell tumors (GCTs) are a class of heterogeneous tumors. The tumors classification system of the World Health Organization classifies them into germinoma and nongerminoma germ cell tumors (NG-GCTs). The later one includes teratoma, embryonal carcinoma, endodermal sinus tumor (yolk sac tumor), chorionic epithelioma (also called choriocarcinoma) and mixed GCTs [1]. But in Europe and North America, GCTs are often classified as secretory type and non-secretory type based on the tumor marker level. Secretory GCTs mean that the level of alpha fetal protein (AFP) in the serum and/or cerebrospinal fluid is more than or equal to 10 ng/dl (or higher than normal value) and/or the level of human chorionic gonadotropin –β (β-HCG) is more than or equal to 50 IU/l (or higher than normal value). The tumor maker level is normal in non-secretory GCTs [2–5]. Pure germinomas are often non-secretory type or accompanying with mild elevation of β-HCG. Elevation of tumor markers is often seen in NG-GCTs except in mature teratoma [6]. Non-secretory pure germinomas are sensitive to radiotherapy/chemotherapy, the long-term survival rate of which is more than 90% for treatment with radiotherapy alone [7]. The dosage and region of radiotherapy for children can be decreased by combined chemotherapy [8–10]. The prognosis of secretory NG-GCTs is worse, as the 5-year overall survival is 10–38% for treatment with radiotherapy alone. Comprehensive treatments such as chemotherapy, radiotherapy or operation are necessary to improve survival rate [7, 11–13]. Therefore, histopathological diagnosis of tumor should be verified before treatment, which is essential for the option of treatment regimen.

Surgical resection of tumor or biopsy are recognized as the most accurate method to determine the pathological property of tumor, the purpose of which is to quickly relieve tumor compression and ease intracranial hypertension without aggravation of neurological dysfunctions and to provide insurance for following radiotherapy, chemotherapy and histological diagnosis [14–18]. However, primary intracranial GCTs are commonly located in the pineal and sellar regions which are deep and have important anatomical structures around. So it is quite difficult and risky to perform surgical resection. Historically, the alternative therapeutic measures such as therapeutic trials of irradiation are commonly used in clinical practice due to complications and mortality rate relevant to surgery [7, 19]. If the tumor has response to low dose of irradiation, such tumor can be clinically diagnosed as germ cell tumor and patients can continue to complete radiotherapy. If patients have no responses to radiotherapy, the diagnosis of NG-GCTs, glioma or other tumors should be considered. In this situation, surgery is also needed to further determine the property of tumor and relieve tumor compression. Although micro-neurosurgery advances, the mortality and morbidity decreased significantly, but mortality rate for midline surgery remains up to 5% [14–15]. For patients who are unable to undertake surgery for pathological diagnosis, what will be the clinical treatment? It is a big puzzle and challenge for both clinicians and patients. This retrospective study collected data of 42 cases (not histologically diagnosed prior to radiotherapy/chemotherapy) clinically diagnosed with primary intracranial GCTs at the Cancer Center of Sun Yat-Sen University and analyzed the clinical effectiveness to provide reference for the treatment of patients with such tumors.

## PATIENTS AND METHODS

### General data of patients

Patients who were clinically diagnosed as primary intracranial GCTs based on clinical symptoms, signs, neuroimaging features and combined analysis of tumor markers but not histologically diagnosed by surgical resection or biopsy prior to the initial radiotherapy (including gamma knife radiosurgery) or chemotherapy were included in this study. From May 2002 to Dec 2015, 42 patients clinically diagnosed with primary intracranial GCTs received chemotherapy and/or radiotherapy at the Cancer Center of Sun Yat-sen University. Patients were assigned to diagnostic chemotherapy group, diagnostic radiotherapy group and gamma knife radiosurgery group based on their initial anti-tumor therapy. The general characteristics of patients were provided in Table 1. Most tumors were found in male, teenagers and located in the pineal and suprasellar regions. The other tumors involved in the basal ganglia region, hypothalamus, callosum, corpora quadrigemina, dorsal thalamus, 3^rd^ ventricle or lateral ventricle. A few patients had disseminated tumors in the ventricular system or spinal cord. The main clinical symptoms include increased intracranial pressure syndrome (dizziness/headache, nausea/vomiting, drowsiness), adjacent cerebral compression syndrome (anorexia/emaciation, insipidus/electrolyte disturbance, impaired vision/vagueness/blindness/diplopia, hearing loss/acousma/tinnitus, ataxia) and endocrine syndrome (sexual precocity/amenorrhea/dysplasia, thyroid hypofunction). The median duration of disease was 3 months from occurrence of symptoms to initial treatment.

**Table 1:** General characteristics of patients clinically diagnosed with primary intracranial germ cell tumors (n=42)

Covariates	Levels	All patients	Treatment group			p-value
			Diagnostic chemotherapy	Diagnostic radiotherapy	Gamma knife radiosurgery	
**Sex**	Male	36 (85.71%)	21 (84.00%)	3 (60.00%)	12 (100.00%)	0.09
	Female	6 (14.29%)	4 (16.00%)	2 (40.00%)	0 (0.00%)	
**Age at initial diagnosis**		16.5 (15.37-19.30)	17.0 (14.67-19.73)	16.0 (11.10-25.30)	16.0 (12.58-21.92)	0.19
**Duration of disease (month)**		3.0 (0.25-25)	3.0 (0.25-25)	12.0 (1-13)	1.0 (0.5-15)	0.15
**Tumor location**	Pineal/suprasellar regions	33 (78.57%)	18 (72.00%)	3 (60.00%)	12 (100.00%)	0.09
Other regions	9 (21.43%)	7 (28.00%)	2 (40.00%)	0 (0.00%)	0.30	
**Stage**	Localized	29 (69.05%)	18 (72.00%)	2 (40.00%)	9 (75.00%)	0.32
	Disseminated	13 (30.95%)	7 (28.00%)	3 (60.00%)	3 (25.00%)	0.29
**Clinical manifestation**						
	Increased intracranial pressure syndrome	21 (50.00%)	11 (44.00%)	4 (80.00%)	6 (50.00%)	0.38
	Adjacent cerebral compression syndrome	34 (80.95%)	20 (80.00%)	4 (80.00%)	10 (83.33%)	0.97
	Endocrine syndrome	7 (16.67%)	5 (20.00%)	1 (20.00%)	1 (8.33%)	0.66
**Secretory tumor**		30 (71.43%)	20 (80.00%)	4 (80.00%)	6 (50.00%)	
	Only elevated AFP	2 (4.76%)	1 (4.00%)	1 (20.00%)	0 (0.00%)	0.45
	Only elevated β-HCG	21 (50.00%)	15 (35.71%)	2 (40.00%)	4 (33.33%)	0.28
	Elevated AFP and β-HCG	7 (16.67%)	4 (16.00%)	1 (20.00%)	2 (16.67%)	0.86
**Non-secretory tumor**		12 (28.57%)	5 (20.00%)	1 (20.00%)	6 (50.00%)	0.15
**Objective response rate**		37 (88.10%)	21 (84.0%)	5 (100.00%)	11 (91.70%)	0.54
**Average survival (month)**		108.7±7.5	114.7±7.5	98.8±21.9	79.4±10.0	
**Median OS (months)**		74.5 (95%CI:62.0-87.8)	80.3 (95%CI:62.4-85.9)	74.6 (95%CI:23.9-133.1)	58.6 (95%CI:40.2-75.4)	
**5-year survival (%)**		77.4%	85.8%	75.0%	63.6%	0.44

### Tumor markers

The β-HCG level (normal value range at this center: 0-3 mIU/ml) and AFP level (normal value range at this center: 0-25 ng/ml) in the serum and cerebrospinal fluid were determined in 42 cases. 30 cases had secretory tumors (elevated tumor markers were found in the serum and cerebrospinal fluid) and accounted for 71.4%, including 2 cases with only elevated AFP (269.4, 662.0ng/ml), 21 cases with only elevated β-HCG (3.31-1805 mIU/ml), 7 cases with elevated AFP (32.9-2195 ng/ml) and β-HCG (4.1-6716 mIU/ml). 12 cases had non-secretory tumors (the tumor markers in the serum and cerebrospinalfluid were normal).

### Therapeutic method

Patients should adequately understand and sign the informed consent prior to diagnostic chemotherapy or radiotherapy. Patients who had severe intracranial hypertension caused by obstructive hydrocephalus were treated with ventricle-peritoneal shunt prior to chemotherapy or radiotherapy. Diagnostic chemotherapy used PEB regimen (Total amount of 80-100mg/m^2^ cisplatin, 300-500 mg/m^2^ teniposide or etoposide was administered in divided doses over 3-5 days. Doses of 10 mg/m^2^ bleomycin were administered on Day 1 and Day 5. Treatment repeated every 3 to 4 weeks). After one course of chemotherapy, patients were reviewed with MRI brain scans. If tumor regression was observed, chemotherapy continued for 4-6 courses in combination with radiotherapy to the brain. Otherwise, treatment was discontinued. Diagnostic radiotherapy used whole-brain radiotherapy. Patients were followed with MRI brain scans after 10-20 Gy of radiotherapy was delivered. If tumor regression was observed, radiotherapy continued until completion (the whole-brain or whole-central-nervous-system (whole-CNS) dose was 30 Gy and the regional dose increased to 50 Gy) with or without chemotherapy of PEB regimen. Otherwise, the treatment was discontinued. Patients in the gamma knife radiosurgery group had received gamma knife radiosurgery at initial diagnosis in other institutes. And they came to our center and received chemotherapy of PEB regimen with or without whole-brain or whole-CNS radiotherapy after they were clinically diagnosed as primary intracranial GCTs.

### Assessment of outcome and adverse reaction

Assessment of outcome complied with response criteria recommended by Macdonald. Complete response (CR): disappearance of all known enhancing and nonenhancing lesions for a minimum of 4 weeks, no appearance of any new lesion, off steroids, and neurologically stable or improved. Partial response (PR): 50% or greater decrease in the sum of the product of bidimensional measurements (the maximum diameter multiplied by the largest diameter at right angles to this) of all measurable lesions maintained for a minimum of 4 weeks, no increase in any lesion, no new lesions, steroids stable or reduced, and neurologically stable or improved. Stable disease (SD): ≥ 25% but <50% decrease in the sum of the product of bidimensional measurements of all measurable lesions for a minimum of 4 weeks, no appearance of any new lesion; steroids stable or reduced, and neurologically stable or improved. Progressive disease (PD): ≥ 25% increase in the sum of the product of bidimensional measurements of all measurable lesions, or any new lesion, or neurologically worse. The overall survival (OS) of patients was defined as duration from the date of initiating chemotherapy or radiotherapy to the date of death or the last follow-up. The assessment of adverse reactions was complied with criteria in CTC version 3.0 (Common Terminology Criteria for Adverse Events v3.0).

### Statistical analysis

Numeric variables were summarized as mean (standard deviation) or median (interquartile range), as appropriate. Categorical variables were reported as counts (percentage). To compare continuous variables (age) that appear to have symmetric distributions among multiple treatment types, analysis of variance was employed. To compare categorical variables among multiple treatment groups, chi-square test or fisher's exact (n<5) were performed. The Kaplan-Meier method was used to draw the survival distributions, and the log-rank test was used to assess difference in survival experience.Median survival time with 95% CI were summarized for each treatment group. All tests of hypotheses were two-sided and conducted at 0.05 level of significance. SPSS17.0 was used in all statistical analyses.

## RESULTS

### Baseline characteristics

Forty-two patients were not histologically diagnosed by resection or biopsy prior to the initial chemotherapy or radiotherapy. Twelve patients with obstructive hydrocephalus were treated with ventricle-peritoneal shunt prior to chemotherapy/radiotherapy. Two patients received resection of residual tumor after chemotherapy/radiotherapy. They were pathologically diagnosed as mature teratoma and mixed GCT, respectively.

### Diagnostic chemotherapy group

Twenty-five patients were initially treated with diagnostic chemotherapy. After chemotherapy, CR was observed in 11 patients. PR was observed in 10 patients. SD was observed in 2 patients. Two patients were not evaluated. The objective response (CR+PR) rate was 84.0%. Ten of 11 patients with CR received adjuvant whole-brain or whole-CNS radiotherapy while 1 (chemotherapy alone, more than 24-month survival) of them did not receive the subsequent radiotherapy. All 11 patients kept alive with CR as of the last follow-up. Ten patients with PR received whole-CNS radiotherapy. After radiotherapy, CR was observed in 5 patients. Further tumor regression was observed in 4 patients. Nine patients kept alive as of the last follow-up. But tumor regression was not observed in 1 patient (185 ng/ml AFP, 66.4 mIU/ml β-HCG). Then this patient received resection of partial tumor and the pathological diagnosis was mature teratoma. The patient died of hypothalamus syndrome and secondary aplastic anemia 2 years after operation (42-month survival). Decrease of tumor markers was not observed in 2 patients with SD after chemotherapy. One (91.2 mIU/ml β-HCG, normal AFP) of them was 4 years old and had PR after treatment with localized radiotherapy (more than 56-month survival). The other one (662ng/ml AFP, normal β-HCG) received surgery and the pathological diagnosis was germinoma mixed with endodermal sinus tumor). After surgery, this patient received whole-CNS radiotherapy (more than 6-month survival). One patient (31 mIU/ml β-HCG) with secretory tumor and 1 with non-secretory tumor did not received follow-up treatment (chemotherapy alone) after one course of chemotherapy. So their responses could not be assessed. Both patients died (4-month and 24-month survivals, respectively).

### Diagnostic radiotherapy group

Five patients were initially treated with diagnostic radiotherapy. After radiotherapy, CR was observed in 3 patients. PR was observed in 2 patients. The objective response rate was 100%. Four of 5 patients received radiotherapy alone (3 cases received whole-CNS radiotherapy and 1 case received whole-brain radiotherapy) and were not treated with follow-up adjuvant chemotherapy. All patients kept alive with CR or PR as of the last follow-up. One patient with PR (906 ng/ml AFP, 4.1 mIU/ml β-HCG) received two courses of adjuvant chemotherapy and further tumor regression was observed. But then the chemotherapy was discontinued due to hemorrhage in the tumor. After 2 months, the tumor was disseminated in the vertebral canal. The patient failed to survive (23-month survival) despite receiving rescue chemotherapy of IEP regimen (IFO, VM-26, DDP).

### Gamma knife radiosurgery group

Twelve patients were treated with gamma knife radiosurgery at initial diagnosis at another hospital. After treatment, CR was observed in 2 patients. PR was observed in 9 patients. SD was observed in 1 patient. The objective response rate was 91.7%. Three patients received adjuvant chemotherapy in our department after gamma knife radiosurgery. Two cases of them received adjuvant chemotherapy followed by combined whole brain or whole-CNS radiotherapy. One case with SD after gamma knife radiosurgery did not receive subsequent radiotherapy. Three cases kept alive (1 case with CR and 2 cases with PR) as of the last follow-up.

Nine cases did not receive adjuvant chemotherapy or routine radiotherapy after gamma knife radiosurgery. After 2 -18 months, tumors were disseminated. Then they came to this center and received rescue chemotherapy of PEB regimen. Seven cases received whole-CNS or whole-brain radiotherapy after rescue chemotherapy while the other 2 cases did not. As of the last follow-up, CR was observed in 5 cases. PR was observed in 1 case and 3 cases died. The details were as follows: 1 case (23.02 mIU/ml β-HCG, normal AFP) who had disseminated tumors after previous multiple gamma knife radiosurgeries died despite rescue chemotherapy (40-month survival, the duration was 18 months from initial gamma knife radiosurgery to initial chemotherapy). One case (43 ng/ml AFP, 6.3 mIU/ml β-HCG) had disseminated tumors in the ventricle 2 months after gamma knife radiosurgery. Then the patient received rescue chemotherapy for 4 courses but refused to continue follow-up radiotherapy. After 9 months, this patient received whole-CNS radiotherapy and was unable to survive (27-month survival). One case (normal tumor markers) had disseminated tumors in the ventricle and spinal cord despite previous multiple gamma knife radiosurgeries. Then this case received rescue chemotherapy for 3 courses and PR was observed. And the case continued to receive whole-CNS radiotherapy and CR was observed. But 5 months after discontinuation of radiotherapy, recurrence occurred with complicated secondary aplastic anemia and hypothalamus syndrome. This patient died eventually (27-month survival, the duration was 14 months from initial gamma knife radiosurgery to initial chemotherapy).

### Survival analysis

**Figure 1:**
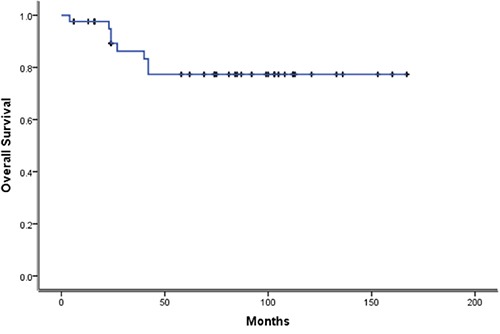
Kaplan-Meier survival Kaplan curves for Overall Survival of 42 cases

**Figure 2:**
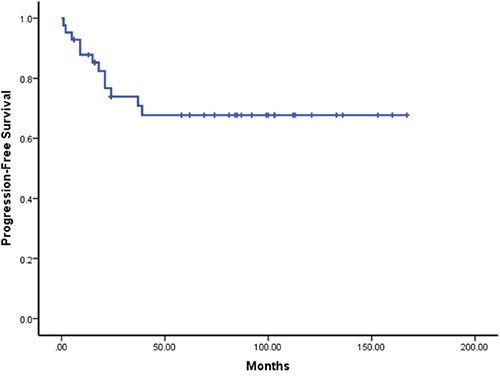
Kaplan-Meier survival Kaplan curves for Progression-free Survival of 42 cases

**Figure 3:**
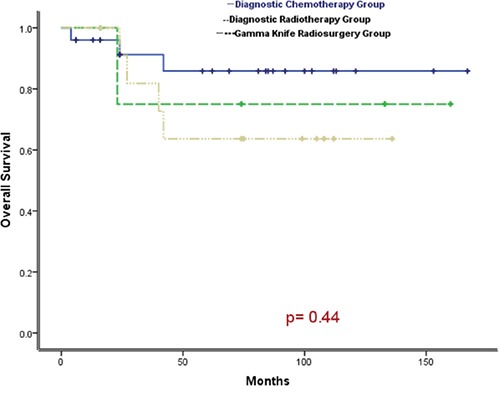
Comparison of survival curves among patients in diagnostic chemotherapy group, radiotherapy group and gamma knife radiosurgery group (P = 0.44)

**Figure 4:**
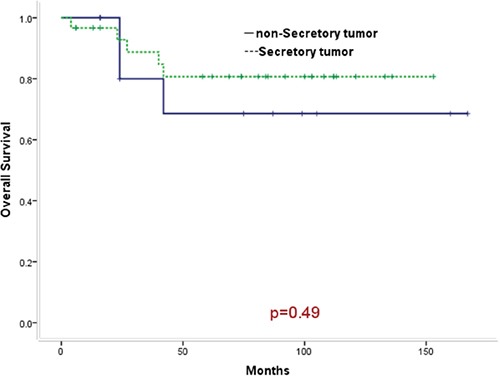
Comparison of survival curves between patients with secretory tumors and patients with non-secretory tumors (P = 0.49)

The median duration of follow-up was 74 months (4–166 months) as of December, 2015. As of the last follow-up, 7 of 42 cases died of tumor progression. Thirty-five cases survived. Among them, 25 cases (59.5%) had CR. 10 cases had PR. The results of comprehensive therapy in all patients were shown in Table 2. The 5-year overall survival and progression-free survival of all patients was 77.4% and 67.7%,respectively, as shown in Figure 1 and Figure 2. The 5-year overall survival were 85.8% for the diagnostic chemotherapy group, 75.0% for the diagnostic radiotherapy group and 63.6% for the gamma knife radiosurgery group, respectively. There were no statistically significant difference (*p* value = 0.44), as shown in Figure 3. The 5- year overall survival were 68.6% for patients with secretory tumors and 80.7% for patients with non-secretory tumors, respectively. There were no statistically significant difference (*p* value = 0.49), as shown in Figure 4.

**Table 2:** Outcome of comprehensive therapy in 42 cases clinically diagnosed with intracranial germ cell tumors

		Secretory type (n=30)				Non-secretory type (n=12)				
	All patients (n=42)	Total	Chemotherapy (n=20)	Radiotherapy (n=4)	Gamma knife (n=6)	Total	Chemotherapy (n=5)	Radiotherapy (n=1)	Gamma knife (n=6)	p-value
**Outcomes of initial treatment**										0.86
CR	16 (38.10%)	11 (36.67%)	8 (40.00%)	2 (50.00%)	1 (16.67%)	5 (41.67%)	3 (60.00%)	1 (100.00%)	1 (16.67%)	
PR	21 (50.00%)	16 (53.33%)	9 (22.50%)	2 (50.00%)	5 (83.33%)	5 (41.67%)	1 (20.00%)	0 (0.00%)	4 (66.67%)	
SD	3 (7.14%)	2 (6.67%)	2 (10.00%)	0 (0.00%)	0 (0.00%)	1 (8.33%)	0 (0.00%)	0 (0.00%)	1 (16.67%)	
NA	2 (4.76%)	1 (3.33%)	1 (5.00%)	0 (0.00%)	0 (0.00%)	1 (8.33%)	1 (20.00%)	0 (0.00%)	0 (0.00%)	
**Comprehensive therapy pattern**										0.15
Radiotherapy+ chemotherapy	23 (54.76%)	20 (66.67%)	19 (95.00%)	1 (25.00%)	0 (0.00%)	3 (25.00%)	3 (60.00%)	0 (0.00%)	0 (0.00%)	
Radiotherapy alone	4 (9.52%)	3 (10.00%)	0 (0.00%)	3 (75.00%)	0 (0.00%)	1 (8.33%)	0 (0.00%)	1 (100.00%)	0 (0.00%)	
Chemotherapy alone	3 (7.14%)	1 (3.33%)	1 (5.00%)	0 (0.00%)	0 (0.00%)	2 (16.67%)	2 (40.00%)	0 (0.00%)	0 (0.00%)	
Gamma knife + radiotherapy + chemotherapy	9 (21.43%)	5 (16.67%)	0 (0.00%)	0 (0.00%)	5 (83.33%)	4 (33.33%)	0 (0.00%)	0 (0.00%)	4 (66.67%)	
Gamma knife + chemotherapy	3 (7.14%)	1 (3.33%)	0 (0.00%)	0 (0.00%)	1 (16.67%)	2 (16.67%)	0 (0.00%)	0 (0.00%)	2 (33.33%)	
**Results of the last follow-up**										0.55
CR	25 (59.52%)	17 (56.67%)	12 (60.00%)	2 (50.00%)	3 (50.00%)	8 (66.67%)	4 (80.00%)	1 (100.00%)	3 (50.00%)	
PR	10 (23.81%)	8 (26.67%)	6 (30.00%)	1 (25.00%)	1 (16.67%)	2 (16.67%)	0 (0.00%)	0 (0.00%)	2 (33.33%)	
Death	7 (16.67%)	5 (16.67%)	2 (10.00%)	1 (25.00%)	2 (33.33%)	2 (16.67%)	1 (20.00%)	0 (0.00%)	1 (16.67%)	

### Adverse reactions

Forty-two patients received chemotherapy of PEB regimen for a total of 142 courses. The major adverse reactions were grade III- IV bone marrow suppression. The incidence was 35.2%. Five cases of them had infection and fever. The symptoms resolved after administration of G-CSF, platelet infusion and anti-infection therapy. The incidence of grade II- III nausea/vomiting was 45.8%. The symptoms relieved after symptomatic treatment. The incidence of gradeI-II elevation of hepatic transaminase was 9.2%. One case had renal impairment (mildly elevated serum creatinine). One case had recurrence after multiple gamma knife radiosurgeries and developed secondary aplastic anemia and hypothalamus syndrome after chemotherapy and radiotherapy. The symptoms did not relieve after administration of symptomatic treatment and hormone replacement therapy. The case died of tumor recurrence after 1 year. Three cases with sustained CR developed hypopituitarism. The growth and mentality returned to normal after hormone replacement therapy.

## DISCUSSION

Primary intracranial GCTs are a class of heterogeneous tumors. Surgical treatment can quickly relieve tumor compression and ease intracranial hypertension which provided insurance for following radiotherapy, chemotherapy and histological diagnosis. However, primary intracranial GCTs are commonly located in the midline regions that have special anatomical structures. So it is quite difficult and risky to perform surgical resection. Although micro-neurosurgery advances, the mortality remains quite high for midline surgery, and thus for some patients unable to be pathologically diagnosed, it is still challenge for treatment dicission [14–18]. According to the basic principles of chemotherapy or radiotherapy against tumor, patients who receive chemotherapy or radiotherapy must be pathologically or cytologically diagnosed as having cancer. When it is a special case in the absence of above evidence, such as in the case of emergency or unable to obtain the pathological tissues, chemotherapy or radiotherapy might be considered under the instruction of experienced expert when adequate clinical evidence shows that the benefits far exceed the risks. This study collected clinical data of 42 cases whom were clinically diagnosed as having primary intracranial GCTs at our cancer center during last 10 years. Patients included in this study were not histologically diagnosed by surgical resection or biopsy prior to the initial radiotherapy (including gamma knife radiosurgery) or chemotherapy. This study retrospectively analyzed the clinical characteristics, treatment method and treatment outcomes, which was aimed at providing reference for the treatment of patients with such tumors.

For secretory tumors, it is not difficult to diagnose primary intracranial GCTs. Chorionic epithelioma and immature tumor cells secrete β-HCG. AFP is secreted by endodermal sinus tumor cells in the immature tumor cells. Germinoma cells secrete PLAP. These tumor markers have become essential indicators of assessment for patients with GCTs before treatment. But they can't differentiate accurate subtype of tissue [2–3]. Thirty patients in this group were clinically verified as having primary intracranial GCTs (secretory tumors; NG-GCTs or pure germinoma with lightly elevated β-HCG were considered) based on clinical symptoms, signs, neuroimaging characteristics combined with the elevation of AFP/β-HCG levels in the serum and cerebrospinal fluid, thereby undertaking diagnostic chemotherapy or radiotherapy. Tumor markers often decreased rapidly in patients who had responses to radiotherapy/chemotherapy. Patients who had no responses were considered to be further pathologically diagnosed by surgical removal of the tumors. Two of 30 cases in this group undertook resection of residual tumors after chemotherapy/radiotherapy. They were pathologically diagnosed as having mature teratoma and mixed GCT, respectively. Literature reported that CNS GCTs could be classified into three categories according to the pathological types and prognosis. Pure germinoma and mature teratoma had good prognosis and the 10-year overall survival were more than 90%. The 5-year overall survival were 70-90.4% for patients with medium prognosis and 9.3%-27.3% for patients with bad prognosis, respectively [19–22]. The 5-year overall survival was 80.7% for 30 cases (only 2 cases received follow-up surgery) with secretory tumors in this group, which was close to the survival rate of CNS - GCTs (pathologically diagnosed by surgery) with medium prognosis reported in literatures.

For the non-secretory tumors, it is difficult to be diagnosed. Non-secretory tumors with normal AFP/β-HCG may be pure germinoma, but also may be mature teratoma, glioma or pineoblastoma and other tumors. Pure germinoma is very sensitive to radiotherapy and chemotherapy, and generally other tumors have no response [2–4, 20]. The 5-year overall survival was 68.6% for 12 cases with non-secretory tumors in this group. As of the last follow-up, 8 in 12 cases had CR and 2 cases had PR while 2 cases died (1 case only undertook chemotherapy alone for a course. It was unable to evaluate response. The survival was 24 months. 1 case had disseminated tumors in the ventricle and spinal cord after gamma knife radiosurgery for many times. Then the case received rescue chemotherapy combined with whole-CNS radiotherapy and CR was observed. But recurrence occurred with complicated secondary aplastic anemia and hypothalamus syndrome. The survival was 27 months). Analysis of the 2 patients who died found that their treatments were not standard, thereby influencing the prognosis.

According to the initial anti-tumor treatments of patients, 42 patients in this group could be classified into 3 groups which are diagnostic chemotherapy group, diagnostic radiotherapy group and gamma knife radiosurgery group. Histological diagnosis of tumors is regarded as the most accurate method. But GCTs have extremely high sensitivity to the irradiation. As a result, in the 1980s, experts in Japan - where GCTs occur frequently - first put forward the concept of “diagnostic radiotherapy” [7, 19]. It has been a decade since “diagnostic radiotherapy” was used to distinguish the nature of GCTs without pathological confirmation. Many medical institutions have used this method so far. Compared with surgery or biopsy, it has virtues such as mild damage, lower risk and less cost. After confirmation of the nature of GCTs by diagnostic radiotherapy, patients can continue to complete radiotherapy and chemotherapy without surgery until recovery. For the highly malignant GCTs (such as choriocarcinoma or endodermal sinus tumor) diagnosed by tumor markers, the “sandwich” therapy is used in some countries [5, 13]. In this method, “diagnostic chemotherapy” is firstly used to observe whether the levels of tumor markers reduced, and then radiotherapy is delivered followed by review with MRI. If there is still residual tumor, resection is performed. Add chemotherapy after surgery. This method obviously improved the survival rate, but generally few patients can survive for a long time. Most patients (30 cases) in this group were diagnosed as having GCTs by elevated tumor markers and received “diagnostic chemotherapy” as initial treatment, and 24 cases in 25 patients in diagnostic chemotherapy group also received adjuvant radiotherapy. But only 1 case in 5 patients in diagnostic radiation group received adjuvant chemotherapy. Using comprehensive treatment pattern of chemotherapy combined with radiotherapy, 5-year overall survival was 85.5% in diagnostic chemotherapy group, higher than 75.0% in diagnostic radiation group. The curves seem parallele and obviously seperated among the 3 groups, which means the OS should be significantly different among groups, but there was no statistically significant difference. We have excluded the potential outliers so the separated curves without significant difference maybe due to the small sample size. As a result, we will collect more cases in the future to certify the difference.

Twelve patients received gamma knife radiosurgery at initial diagnosis at another hospital. The short-term objective response (CR + PR) rate was as high as 91.7%. Three patients received adjuvant chemotherapy with or without conventional radiotherapy in our center after gamma knife radiosurgery. As of the last follow-up, all kept alive (1 case with CR, 2 cases with PR). But 9 patients did not receive adjuvant chemotherapy or conventional radiotherapy after gamma knife radiosurgery and they had disseminated and metastatic tumors after 2-18 months. Then they came to our center and undertook rescue chemotherapy of PEB regimen with or without conventional radiotherapy. As of the last follow-up, 5 cases had CR and1 case had PR while 3 cases died. 1 case of them had complicated secondary aplastic anemia and the hypothalamus syndrome, and eventually died of tumor recurrence and progression. The 5-year overall survival was 63.6% for 12 cases in the gamma knife radiosurgery group, lower than those in the diagnostic chemotherapy and radiotherapy groups. But there was no statistically significant difference. Gamma knife radiotherapy can pinpoint the tumor cells and kill them to reduce the damage to the surrounding normal tissues. This advantage of gamma knife radiosurgery is taken by people to treat brain tumors to reduce the influence on the hypothalamus - pituitary axis, especially for children and adolescents with germ cell tumors. However, malignant germ cell tumors in the brain are invasive and metastatic, and often spread to the brain, ventricle and spinal cord along the channels of ventricle and brain spinal cord. High recurrence and metastasis rate are observed in patients with malignant germ cell tumors in the brain and treated with gamma knife radiosurgery alone. Patients who have recurrence and metastasis after gamma knife radiosurgery have difficulty in receiving treatment again and receiving following radiotherapy, especially for patients who have received the gamma knife radiosurgery for many times. If these patients receive standard treatment as initial treatment, the survival rate should be higher compared with patients receiving gamma knife radiosurgery alone. Gamma knife radiosurgery should be combined with other therapeutic methods as part of comprehensive treatment [23].

In terms of adverse reactions, the main adverse reactions of chemotherapy of PEB regimen are bone marrow suppression and nausea/vomiting. But they can be controlled after administration of G-CSF and blood transfusion, anti-nausea and symptomatic treatment. It is noticeable to carefully assess benefits and risks of chemotherapy and radiotherapy for patients and avoid the secondary aplastic anemia, hypothalamus syndrome and other serious complications when rescue chemotherapy and radiotherapy are considered in patients who have recurrence after previously receiving gamma knife radiosurgery for many times.

In conclusion, primary central nervous system germ cell tumors are a group of heterogeneous tumors. Tumor resection or biopsy is recognized as the most accurate method to determine the pathological property of tumor. But for some patients who can not be pathologically diagnosed, the clinical diagnosis can be made based on clinical symptoms, signs, neuroimaging characteristics combined with analysis of the tumor markers. When patients are clinically diagnosed as having primary intracranial GCTs, they can receive comprehensive treatments such as chemotherapy combined with radiotherapy and some of them can still have good responses.
